# Dissecting the Potential Interplay of DEK Functions in Inflammation and Cancer

**DOI:** 10.1155/2015/106517

**Published:** 2015-09-06

**Authors:** Nicholas A. Pease, Trisha Wise-Draper, Lisa Privette Vinnedge

**Affiliations:** ^1^Division of Oncology, Cincinnati Children's Hospital Medical Center, Cincinnati, OH 45229, USA; ^2^Department of Internal Medicine, Division of Hematology/Oncology, University of Cincinnati College of Medicine, Cincinnati, OH 45267, USA

## Abstract

There is a long-standing correlation between inflammation, inflammatory cell signaling pathways, and tumor formation. Understanding the mechanisms behind inflammation-driven tumorigenesis is of great research and clinical importance. Although not entirely understood, these mechanisms include a complex interaction between the immune system and the damaged epithelium that is mediated by an array of molecular signals of inflammation—including reactive oxygen species (ROS), cytokines, and NF*κ*B signaling—that are also oncogenic. Here, we discuss the association of the unique DEK protein with these processes. Specifically, we address the role of DEK in chronic inflammation via viral infections and autoimmune diseases, the overexpression and oncogenic activity of DEK in cancers, and DEK-mediated regulation of NF*κ*B signaling. Combined, evidence suggests that DEK may play a complex, multidimensional role in chronic inflammation and subsequent tumorigenesis.

## 1. Introduction

Chronic inflammation has been linked to cancer for decades with several epidemiologic reports suggesting causation. In fact, several infectious and noninfectious known causes of cancer, such as viral infection (human papilloma virus, HPV, and Epstein Barr virus, EBV),* Helicobacter pylori* infection, smoking, and asbestos exposure to name a few, can induce inflammation prior to tumor formation. Additionally, upregulation through polymorphisms of the proinflammatory cytokines, tumor necrosis factor (TNF), and interleukin-1 (IL-1) has been associated with poor prognoses and disease severity in non-Hodgkin's lymphoma and gastric cancer, respectively [1, 2]. Conversely, administration of known anti-inflammatory medications and herbs such as nonsteroidal anti-inflammatory drugs (NSAIDs), curcumin, and ginseng has been associated with a decreased risk of cancer, leading to several clinical studies investigating these agents as possible adjunct treatments [3–5]. However, the exact mechanisms of causation have remained unclear and in some instances, anti-inflammatory medications have been associated with a higher risk of cancer, making the association more complicated than initially proposed [6].

In general, inflammation and innate immunity are felt to be protumorigenic whereas adaptive immunity is antitumorigenic. In fact, tumor associated macrophages (TAMs), which are recruited during the inflammatory response, are indicators of a poor prognosis when identified in tumor tissue [7] while high levels of cytotoxic T (CD8+) cells, as part of adaptive immunity, correlate with a good prognosis [8, 9]. However, the presence of T cells in the microenvironment of tumors alone may not be sufficient to confer an immune response as they often are not active in recognizing the tumor as nonself. Therefore, a focus on negative regulators of the immune system, such as T regulatory (Treg) cells and other inhibitory molecules, is under investigation as a possible explanation for tumor immune escape. Multiple immune checkpoint molecules such as cytotoxic T lymphocyte antigen-4 (CTLA-4) and “programmed death-1” (PD-1) and its ligands (PD-L1 and PD-L2) are upregulated during the immune response in an attempt to prevent autoimmune damage to normal tissue. However, PD-1 is also induced on T cells after activation by immune stimulation, either by infection or by tumor progression and, therefore, is felt to be a mechanism of immune resistance. Additionally, high levels of PD-1 in the tumor microenvironment and PD-1 ligand (PD-L1) expression have been found in many tumors and are correlated with poor prognoses in multiple tumor types [10–13]. Targeting of these pathways has proved to be exciting and effective, resulting in FDA approval of several agents (ipilimumab, nivolumab, and pembrolizumab) for melanoma with expectations for approval for other tumors in the near future [14–16]. However, not all tumors respond to these immune therapies, requiring a better understanding of the mechanism of failure and the complex interactions of the immune response to tumors.

Several proinflammatory cytokines and chemokines such as IL-1, IL-6, TNF, and IL-8 are often upregulated in response to cancer and are associated with tumor development and progression in mice [17]. Interestingly, it has been demonstrated that the classical IKK-*β*-dependent NF*κ*B pathway may be the link to inflammation and cancer as activation results in upregulation of proinflammatory cytokines as well as several antiapoptotic factors [18]. Targeting the NF*κ*B pathway may, therefore, be another promising approach to antitumor therapy either alone or in combination with traditional therapies or checkpoint blockade.

## 2. DEK Structure and Functions

One protein recently found to control NF*κ*B activity is the DEK oncogene. DEK is a highly conserved chromatin-associated, nonhistone phosphoprotein (43 kDa) that was originally identified as part of a fusion protein (DEK-CAN) in an acute myeloid leukemia (AML) subtype with translocation t(6;9), a balanced translocation, which confers a poor prognosis [19–21]. The presence of DEK* in vitro* was originally noted for its ability to partially correct cell sensitivity to mutagens and radiation in ATM-deficient fibroblasts [22]. Although it has no known enzymatic activity nor known homologs, it dynamically interacts with RNA, DNA, chromatin, and associated proteins to alter transcription, mRNA processing, DNA replication and repair, and chromatin topology [23–27]. DEK has three DNA binding domains: a central SAF-box, pseudo-SAF/SAP-box, and a C-terminal unique binding domain. The SAF-box domain enables DEK to preferentially bind cruciform and four-way junction structures and induce positive supercoiling; however, the C-terminal binding domain can facilitate DNA-DEK-DEK interactions that may facilitate regulatory processes [20, 28–30].

Nonetheless, other DEK domains participate in important molecular functions. Specifically, the acidic domains of DEK bind chromatin-bound histones and prevent optimal PCAF and p300-mediated histone acetyltransferase activity (HAT). This causes hypoacetylation of DEK-bound regions of nucleosomes and can result in inhibition of HAT-mediated transcriptional activation [31]. Also, the physical interaction between DEK with Daxx and HDACII can assist in transcriptional repression by promoting histone deacetylation [25]. Additional structure-function studies have identified the SAP domain for its importance in DEK function. When the SAP domain of DEK interacts with casein kinase 2 (CK2) in the presence of ATP, DEK is phosphorylated. This DEK-CK2 complex displays an affinity for histone H3.3, a histone variant associated with active chromatin, and limits its placement on chromatin by protecting it from other potential histone chaperones that redistribute H3.3 in a DAAX/ATRX-dependent manner from PML nuclear bodies [32, 33]. Furthermore, DEK is also necessary for optimal binding of heterochromatin protein 1-*α* (HP1-*α*) to the repressive chromatin mark, H3K9me3. This interaction facilitates silencing loops that prevent histone acetylation and protect heterochromatin integrity [34]. Finally, DEK binding to chromatin was found to limit access of the transcriptional machinery to chromatin, which could be disrupted by PARP1 and another histone chaperone, SET [35]. These functions suggest that DEK has the capacity to modify chromatin, via regulating histone acetylation and placement, in a manner that silences the expression of particular regions while also promoting general genomic stability.

However, it is also worth mentioning that some reports indicate that DEK can function as a positive transcriptional cofactor to induce gene expression [33, 36, 37]. In* Drosophila*, DEK was associated with more transcriptionally active regions of chromatin and coactivated the nuclear ecdysone receptor, promoting its functions as a transcriptional activator [33]. In murine breast tumor models, DEK also drives expression of Wnt ligands, resulting in the promotion of *β*-catenin transcriptional activity, which has also been noted in human breast cancer cells [38, 39]. Chromatin immunoprecipitation sequencing (ChIP-Seq) results have also revealed that DEK preferentially binds areas of euchromatin near transcription start sites of highly expressed genes, many of which include motifs for common transcriptional regulators such as SP1 and RNA polymerase II [37].

Although the specific physical interactions are unknown, the molecular functions of DEK also extend to roles in DNA damage and stress response. DEK expression is necessary for proper DNA-PK mediated recruitment of DNA damage repair proteins Ku70/80 during nonhomologous end-joining (NHEJ) and prevents DNA damage accumulation that results in ATM mediated apoptosis [40]. This also supports evidence of DEK complementation in ataxia-telangiectasia cells, in which DEK fragments remedied the DNA damage phenotypes of ATM deficient fibroblasts [22]. DEK overexpression can also cause the destabilization of p53, resulting in the inhibition of normal p53-dependent apoptosis in cancer cells [41]. DEK also has a role in preventing p53-independent apoptosis by promoting the transcription of* MCL-1*, an antiapoptotic member of the BCL-2 family [42].

The molecular functions of DEK can be regulated by an array of protein modifications. Phosphorylation of DEK by CK2 weakens DEK-DNA binding [43] and has been linked to the secretion of DEK [44]. Other modifications that change DEK localization and function include acetylation by p300 [45], poly(ADP-ribosyl)ation by PARP1 [46], and truncation by DPP4 [47]. These posttranslational modifications of DEK are crucial for understanding the molecular functions that could contribute to pathogenesis. Phosphorylation by CK2 and poly(ADP-ribosyl)ation by PARP1 can induce the release of DEK from chromatin, enabling it to function beyond chromatin remodeling [35]. Nonchromatin bound DEK can contribute to mRNA splicing events by interacting with the serine/arginine repeats of splicing complexes, while phosphorylation of DEK serines enables it to associate with U2AF and facilitate intron excision; there is evidence that the role of DEK in mRNA splicing may play a role in alternative splicing events of transcripts from genes such as tropomyosin* TPM1* [48–51]. The function of DEK in the cytoplasm, if present, has not been determined; however, there are several different functions for DEK as a secretory molecule. These include inducing white blood cell migration as a chemoattractant [36], interacting with anti-DEK antibodies that trigger autoimmune responses [52], and possibly promoting chromatin remodeling and prosurvival functions by being taken up by neighboring cells [53]. Interestingly, it is currently unknown what conditions induce the posttranslational modifications of DEK that result in its delocalization from chromatin and secretion, resulting in pathological activities.

The ubiquitous and pleiotropic nature of DEK mandates that the expression and modification of DEK are tightly regulated in order to avoid pathology. The dysregulation of DEK can disturb normal cell functions and potentiate pathogenesis resulting in transformation, chemoresistance, inflammation, and tumor development. In this review, we postulate that DEK may be a crucial link between inflammation and tumorigenesis. We will discuss the role of DEK in viral infection and epitope presentation, which may provide mechanisms for both promoting intracellular viral oncogenesis and for eliciting T cell mediated immune responses that induce proinflammatory cytokines. These cytokines, such as IL-8, can further contribute to chronic inflammation, promote growth signaling in neighboring cells, and stimulate DEK secretion by macrophages. As a secretory molecule, DEK can be recognized by anti-DEK antibodies or be taken up as a functional exogenous protein by neighboring cells. The first function can exacerbate chronic inflammation and induce more proinflammatory factors that create favorable tumor microenvironments. The second secretory DEK function allows for excess DEK to amplify its normal intracellular, often prooncogenic, functions such as chromatin remodeling, transcriptional repression/activation, DNA damage repair, promoting cell proliferation, and silencing apoptotic pathways. These cellular consequences also have been observed when DEK expression is transcriptionally upregulated within a cell by other mechanisms. One potentially intracellular oncogenic function of DEK is its role as a transcription cofactor for NF*κ*B activity. This regulation of the NF*κ*B signaling pathway, as well as the other chromatin modifying and cell signaling roles of DEK, may provide a mechanistic link between inflammation and tumorigenesis.

## 3. DEK Expression and Function during Tumorigenesis and Inflammation

As a well-established oncogene, DEK overexpression has been documented in a continually expanding list of malignant neoplasms, including hepatocellular carcinoma, brain cancer, bladder cancer, retinoblastoma, T cell large granular lymphocytic leukemia, breast cancer, cervical cancer, melanoma, chronic lymphocytic leukemia, colon cancer, head and neck squamous cell carcinomas, and prostate cancer [42, 54–65].* DEK* overexpression is most frequently caused by aberrant transcription via E2F [66], YY1, NF-Y [67], and ER-*α* transcription factors [68]. Increased* DEK* copy number as a result of gains on 6p22 is also observed in bladder cancer and retinoblastoma [57, 69]. DEK protein degradation can be induced by SPOP and FBXW7-alpha ubiquitin ligases, both of which are tumor suppressors and frequently experience loss-of-function mutations in cancers [49, 65, 70]. High DEK expression, and the presence of the DEK-CAN fusion gene, often correlates with higher grade, aggressive tumors [42, 71], chemoresistance [42, 60, 68, 72, 73], invasion [38, 39, 60], and poor patient prognosis [74–78]. DEK may contribute to these oncogenic activities by an array of different molecular mechanisms. In keratinocytes, DEK overexpression can inhibit senescence and apoptosis by promoting p53 destabilization [41, 79]. DEK overexpression also promotes keratinocyte proliferation while delaying differentiation and can contribute to keratinocyte transformation whereas the DEK-CAN fusion also induces transformation in hematopoietic stem cells [80–82]. In breast cancer cell lines, DEK overexpression promotes cell growth and mobility by inducing *β*-catenin nuclear translocation and enhances tumor growth and metastasis by activating Wnt/*β*-catenin autocrine and paracrine signaling loops [38, 39]. DEK depletion in transformed epithelial cells results in DNA damage, senescence, and apoptosis and can reduce ΔNp63 mediated cell growth [38, 41, 64, 82]. *Dek* ^−/−^ mice also demonstrate greatly diminished tumor formation, growth, and metastasis in both genetic and chemically induced tumorigenesis models [39, 64, 82]. Given its numerous functions in cancer cells, it is no surprise that DEK expression could be used as a biomarker for colorectal and bladder cancers and possibly other solid tumors as well [63, 83]. Of future clinical importance, RNA interference-mediated loss of DEK expression causes dramatic apoptosis or senescence of cancer cells whereas differentiated and nontransformed cells remain relatively unharmed [41, 79, 82].

In addition to gene amplification and overexpression in cancers, DEK expression and secretion are also induced in response to inflammation. In BEAS-2B human bronchial epithelial cells,* DEK* mRNA was upregulated in response to exposure to TiO_2_ particles, which are fine particles found in industrial workplaces that are known to cause airway inflammation and respiratory symptoms in both acute and chronic exposure situations [84]. In addition, microarray analyses of livers from rats fed crude fish oil, which contained high levels of persistent organic pollutants, showed moderately elevated* DEK* expression [85]. In rodents, prolonged exposure to persistent organic pollutants has been shown to cause insulin resistance and was associated with chronic low-grade inflammation [85, 86]. Although the molecular mechanism for this transcriptional regulation is unknown,* DEK* upregulation in response to inflammatory signals is supported by the presence of multiple putative AP-1 (c-Fos/c-Jun), Ets-1, NF-AT, NF*κ*B, STAT4, and C/EBP-*β* transcription factor consensus binding sites in the* DEK* promoter, which are known downstream transcription factors induced by proinflammatory signals (data not shown) [87–89]. Furthermore, secretion of phosphorylated DEK by monocyte-derived macrophages (MDM) is induced by the proinflammatory chemokine interleukin-8 (IL-8) where it becomes a chemotactic factor, attracting neutrophils, CD8+ T lymphocytes, and natural killer cells [44]. Immunosuppressive agents, such as dexamethasone and cyclosporine A, could block the secretion of DEK in MDM cells. This suggests that* DEK* expression, modification, and secretion are induced during inflammation, possibly to mediate cell survival, transcriptional responses, and/or migration of immune cells, which can ultimately result in transformation due to the intracellular oncogenic functions of DEK.

## 4. The Role of DEK during Infection with Cancer-Associated Viruses

Viral infection results in an inflammatory response and many cancers are known to be driven by oncogenic viruses. Examples of cancers linked to viral infection include cervical and other anogenital cancers, oropharyngeal carcinomas, hepatocellular carcinomas, Kaposi's sarcomas, lymphomas, and T cell leukemia. In many instances, oncogenesis is thought to result from persistent, latent infections in which cell signaling processes are perturbed by either viral proteins or the chronic activation of inflammatory processes.

Cervical cancer and, more recently, head and neck cancer have been found to be associated with human papillomavirus (HPV) infection [90, 91]. Although HPV infection is quite common, in many individuals, the immune system clears the virus. However, in a select few, viral infection becomes persistent likely through the inability of infected cells to present antigenic epitopes to the host's adaptive immune system [92]. Upon additional multiple mutations and carcinogenic events often linked to the viral life cycle, some of these chronically infected individuals will develop epithelial carcinomas. Although in normal HPV infection viral DNA remains episomal, in cancer, HPV is often found to be integrated into the host DNA. Integration leads to loss of the normal viral repressor HPV E2 resulting in uninhibited expression of the viral oncogenes E6 and E7. HPV E6 causes degradation of the tumor suppressor p53 while HPV E7 causes inhibition of the retinoblastoma (Rb) family of proteins, effectively halting major tumor suppressor pathways. Expression of HPV E6 and HPV E7 is required for maintenance of the malignant phenotype. Interestingly, DEK was found to be upregulated by HPV E7 and the suppression of DEK in HPV infected cells resulted in senescence [79, 93]. Additional studies demonstrated that* DEK* was an E2F transcription factor target gene, explaining its upregulation in response to retinoblastoma protein inhibition by E7 [66].* Dek* knockout (*Dek* ^−/−^) mice are resistant to HPV E6 and HPV E7 driven squamous cell carcinomas, supporting a critical role for DEK function in HPV-induced tumors [64]. Furthermore, DEK mRNA and protein upregulation are present in both cervical and head and neck cancer specimens, further supporting the importance of continued DEK expression in these cancers [59, 79, 94]. Even more intriguing is that although HPV tumors often carry a higher metastatic potential, HPV+ head and neck cancers confer a better prognosis than their HPV− counterparts, due to enhanced responses to treatment [95, 96]. Some have argued that the adaptive immune response associated with HPV infection is the reason for better responses to therapy [97].

Similar to HPV infection,* DEK* expression is also differentially regulated during EBV infections [98].* DEK* was one of three genes differentially regulated across two EBV+ tumor types and also differentially regulated between nasopharyngeal carcinoma cells with latent and recurrent EBV infections.* DEK* expression was downregulated in recurrent EBV-infected cells but upregulated in latent EBV-infected nasopharyngeal carcinoma (NPC) cells. This provides evidence for* DEK* as a potential viral oncogenic mediator that links EBV latency-reactivation dynamics and cell transformation [98]. This may be the result of a well-documented latent infection response mediated by the Rb-controlled activity of E2F, a known activator of* DEK* expression [66, 99]. This CDK2-Rb/E2F-*DEK* pathway may be a crucial step in EBV-associated transformation of epithelial cells as seen in EBV+ NPC. Furthermore, small DNA tumor viruses, like HPV and EBV, exhibit a common molecular mechanism to inhibit the Rb family of proteins, especially pRb, to drive cellular proliferation, viral replication, and eventually oncogenesis. Therefore,* DEK* upregulation is likely a common event in virally induced tumors.

In addition to being transcriptionally regulated in response to viral infection, DEK also controls the use and maintenance of viral genetic material during human immunodeficiency virus (HIV) and Kaposi's sarcoma-associated herpesvirus (KSHV) infections. Although HIV is not oncogenic, the immune suppression and chronic inflammation it causes dramatically increases the risk of cancer due to coinfection with oncogenic viruses like KSHV, EBV, Hepatitis B and C viruses, and HPV. In addition to binding eukaryotic chromatin, DEK also has unique binding properties that facilitate the use or maintenance of viral genetic material. In the case of HIV, DEK can bind to specific sequences of HIV-2 enhancer regions. These sequences, peri-ets (*pets*) sites, are one of several different* cis*-acting elements of the HIV-2 enhancer that stimulates transcription of viral genes in activated T lymphocytes. Within the HIV-2 enhancer region, the* pets* site contains a TTGGTCAGGG sequence that is found between the two Elf-1 binding sites, PuB1 and PuB2 [100]. DEK specifically binds these* pets* sites in human T lymphocytes, suggesting that DEK can regulate HIV-2 transcription and may be a downstream effector of T cell receptor activation [24]. Further investigation revealed that, upon phorbol ester 12-O-tetradecanoylphorbol-13-acetate (TPA) treatment to activate T cells, DEK is replaced on the* pets* site, in a protein phosphatase-2A (PP2A) dependent manner, with another factor to induce HIV-2 promoter activation. The process is stymied by PKC inhibitors and/or PP2A inhibitors (such as Okadaic acid), suggesting that PKC mediates the catalytic activity of PP2A, which alters the stability or DNA-binding activity of DEK, possibly via dephosphorylation. This change activated HIV-2 LTR and promotes HIV-2 transcription, assisting in the maintenance of HIV-2 infections [101]. However, it is unclear if the same mechanism exists in HIV-1 infected cells because Okadaic acid, which permits DEK retention on the* pets* sites and inhibits HIV-2 transcription, actually activates HIV-1 transcription.

While the presence of DEK in HIV-infected cells primarily controls viral transcriptional activity in T lymphocytes, the presence of DEK in two herpesvirus family infections (EBV and KSHV) has more implications on the occurrence of viral oncogenesis. In both cases, the virus must maintain genetic material during latency but also ensure that viral genomes are passed during mitosis. In KSHV infections, the latency-associated nuclear antigen (LANA) facilitates the association between mitotic chromosomes and viral genomes so that viral genomes are distributed to host daughter cells during latent infections [102]. Two studies have documented LANA-DEK binding that could have implications on KSHV infections and associated oncogenesis. Verma et al. demonstrated that DEK interacts with LANA* in vitro* [103]. Through GST affinity and immunoprecipitation assays, Krithivas et al. determined that DEK binds to the C-terminus of LANA and that a GFP-DEK fusion protein can be seen specifically localized to chromosomes of mouse cells [102]. These studies suggest that DEK-LANA interactions provide a secondary tethering opportunity for KSHV genomes that enable KSHV latency and DEK-driven oncogenesis. Combined, DEK is an important cellular protein that can regulate the transcription and retention of viral genomes while promoting proliferation to facilitate the viral life cycle. Nonetheless, it is unclear what links these persistent viral infections that require or increase DEK expression and the host's inflammatory responses to the viruses.

## 5. DEK Is an Autoantigen in Inflammatory Autoimmune Diseases and Cancer

Nearly two dozen autoimmune diseases have been correlated with increased risk for cancer [104]. DEK autoantibodies have been found in the serum and synovial fluid of patients with many different autoimmune disorders including juvenile idiopathic arthritis (JIA), systemic lupus erythematosus (SLE), sarcoidosis, and rheumatoid arthritis [52]. JIA, formerly juvenile rheumatoid arthritis, is characterized by chronic inflammation in one or more joints and is the most common childhood rheumatoid-related condition [105]. SLE primarily affects women and is characterized by severe inflammation that is believed to be caused by a type I interferon mediated positive feedback loop with active B and T lymphocytes [106]. Sarcoidosis usually occurs in the lungs of patients suffering with this systemic granulomatous disease that is characterized by noncaseating granulomas that result from persistent inflammation of unknown origins [107].

DEK was first described as an autoantigen in JIA patients when the presence of DEK specific antibodies correlated with different subtypes of the condition, most frequently seen in pauciarticular onset JIA in 77% of tested patients. The presence of DEK antibodies in JIA patient serum and synovial fluid was later confirmed by several other groups; one revealed a similar percentage of anti-DEK(+) JIA patients at 57% [108, 109]. In addition to anti-DEK autoantibodies, which are produced by B cells, T cells may also become falsely activated in autoimmune diseases through the presentation of DEK peptides by HLA-A molecules. Specific DEK amino acid sequences (72–80, 163–171, and 155–153) can bind the HLA-A∗0201 subclass associated with the pauciarticular subtype of JIA. This suggests that DEK may form complexes with class I MHC molecules which may provide a mechanism by which antigen presenting cells induce CD8+ stimulation to elicit inflammation events seen in JIA patients [110]. This is further supported in patients with the correlation between positivity for DEK antibodies and the presence of the class I HLA-A2 allele [108]. Furthermore, DEK can also be secreted by synovial macrophages, further compounding inflammatory pathogenesis. The C-terminal region of the secreted form of DEK, which is often acetylated, is recognized by IgG2 antibody complexes. These interactions demonstrate a second potential role for DEK in IgG-complement activation in the mediation of immune responses [111]. Additionally, multiallelic marker genotyping and SNP genotyping revealed that the 3′ UTR of* DEK* was associated with rheumatoid arthritis susceptibility, further supporting evidence that DEK may be a crucial component of arthritis related chronic inflammation [112].

Several early studies discovered anti-DEK antibodies in the serum of patients with SLE and/or sarcoidosis [108, 113, 114]. Wichmann et al. found that 10.4% of tested SLE patients had DEK specific autoantibodies in their serum. The presence of the anti-DEK antibodies was associated with older patients and fewer cutaneous manifestations [115]. Dong et al. provided a broad screening of sera from patients with an array of inflammation-related conditions. They identified elevated frequency of anti-DEK positivity not only in JIA, SLE, sarcoidosis, and rheumatoid arthritis patient sera but also in systemic sclerosis, polymyositis, and tuberculosis patient sera [52]. These studies illuminate the potentially broad role of DEK in inflammation-related functions and interactions during infection and immune responses. However, the role of DEK in these interactions can also have substantial implications in cancer biology and tumor microenvironments.

The multifunctionality of DEK in immune cells and cancer cells suggests a paradoxical outcome in tumor biology. As previously discussed, elevated DEK can promote oncogenic activities in infected and uninfected cells; however, DEK also displays an affinity for inducing immune responses in local areas of expression. There are several mechanisms by which DEK may mediate inflammation and tumor immunity responses in tumor microenvironments. These include (1) transcriptional regulation of antigen presenting molecules [116], (2) stimulation of T cells by epitope presentation [117, 118], and (3) secretion into extracellular matrix [44, 53]. First, DEK has the potential to regulate class II MHC expression by interacting with NF-Y and binding Y-box promoter elements unique to MHC class II alleles [116]. This role as a transcriptional regulator could influence the presentation of tumor-related antigens to CD4+ T cells and thus contribute to adaptive immune responses targeting tumor cells. Second, DEK may be a tumor-associated antigen. Dendritic cells loaded with DEK-CAN AML associated fusion proteins can present DEK epitopes via class II MHC molecules and stimulate specific CD4+ T-cells in coculture [118]. The capacity of DEK to stimulate CD8+ T cells was also documented* in vivo* and* in vitro* [117]. In this study,* DEK* was the only oncogenic transcript identified multiple times in a screening of genes possibly involved in an immune response against neuroblastoma. In subsequent experiments, mice received a T cell stimulant and a Treg inhibitor to enable self-antigen specific immune responses.* In vivo*, this combination increases DEK-specific IgG antibodies found in the serum.* In vitro*, CD8+ T cells from these mice showed elevated activity when cocultured with DEK-loaded macrophages or neuroblastoma cells. Together these three studies implicate DEK as a tumor-associated antigen that may mediate interactions between lymphocytes and tumor cells. Third, and finally, DEK secretion by macrophages also has two major implications on potential tumor microenvironments. As a proinflammatory chemoattractant, secreted DEK can stimulate white blood cell migration, including neutrophils, CD8+ lymphocytes, and natural killer (NK) cells [44]. The implications for this activity in the context of tumorigenic microenvironments are poorly understood. While tumor-associated macrophages and neutrophils are primarily known to promote tumorigenesis [119], CD8+ T cells and NK cells are likely antitumorigenic [120, 121]. Secreted DEK can also be internalized by DEK-deficient HeLa cells, in a heparan sulfate-dependent process, where it can function as a nuclear oncoprotein and rescue DEK depletion-induced DNA-damage repair and heterochromatin integrity [53]. Interestingly, macrophages are not the only cells to secrete DEK; conditioned media from HepG2 hepatocellular carcinoma cells were also found to contain DEK peptides [122, 123]. These results illuminate the potential role for extracellular DEK to stimulate tumor-associated immunological responses and promote intracellular oncogenic activity in neighboring epithelial cells within the tumor microenvironment.

## 6. DEK Regulates NF*κ*B Transcriptional Activity

Through multiple mechanisms, DEK can regulate the activity of numerous oncogenic signal transduction pathways. These include p53 family members, p53 and ΔNp63, to inhibit apoptosis and promote proliferation, respectively, Wnt/*β*-catenin signaling to drive proliferation and invasion, Rho signaling to promote migration, mTOR activity to enhance cellular proliferation, and the NF*κ*B pathway [38, 39, 41, 64, 124–127]. The nuclear factor kappa-light-chain-enhancer of activated B cells (NF*κ*B) family of transcription factors regulates gene expression in response to a variety of external stress and inflammatory stimuli. The NF*κ*B family includes RelA (p65), RelB, c-Rel, p100/p52 (NF*κ*B2), and p105/p50 (NF*κ*B1). These transcription factors are activated as a result of environmental stimuli that include cytokines like tumor necrosis factor alpha (TNF*α*), markers of microbial infection like lipopolysaccharide (LPS), T cell and B cell antigen receptors, and genotoxic stress including radiation and reactive oxygen species. The acute presence of these stress signals rapidly activates cell surface receptors, which eventually result in the activation of I*κ*B kinase (IKK2) in a NEMO-dependent mechanism. IKK then phosphorylates I*κ*B, which triggers its ubiquitination and degradation. The degradation of I*κ*B thus releases its inhibitory binding of NF*κ*B/RelA, permitting the nuclear translocation of the transcription factor complex, where it then binds to transcription cofactors to direct gene expression. In response to these acute stress stimuli, NF*κ*B/Rel family members transcribe genes such as growth factors, inhibitors of apoptosis, and cytokines, primarily through RelA:RelA, c-Rel:p50, and RelA:p50 dimers [128]. This canonical NF*κ*B pathway thus promotes cellular proliferation, inflammation, and immunity to survive the environmental stress. In contrast, the noncanonical NF*κ*B pathway utilizes a NEMO-independent kinase complex that includes IKK1 and NF*κ*B-inducing kinase (NIK) to respond to sustained developmental signals. This noncanonical pathway primarily utilizes RelB:p50 or RelB:p52 complexes, although RelA:p50 dimers may also be involved, to cause cell differentiation during development [129].

NF*κ*B signaling is a crucial pathway involved in both inflammation and tumorigenesis, which is underscored by the finding that patients with chronic inflammatory conditions have an increased risk for developing cancer. The prosurvival functions of NF*κ*B signaling promote tumor cell viability whereas the cytokines that are produced by NF*κ*B transcriptional activity will alter the antitumor immune response. Furthermore, NF*κ*B activity can promote angiogenesis and metastasis and has implications for genome stability [130]. Thus, the proproliferative and prosurvival canonical NF*κ*B signaling pathway may be oncogenic if constitutively activated, which can occur through either activating mutations within the pathway or chronic exposure to cytokines from tumor associated macrophages within the microenvironment [130, 131]. However, the prodifferentiation function of NF*κ*B signaling may be tumor suppressive. In fact, the oncogenic versus tumor suppressive functions of NF*κ*B signaling may be context- and tissue-specific. For example, activated NF*κ*B signaling has been documented in lymphoid malignancies and inflammation-associated colon cancer and other solid tumors. However, inactivated NF*κ*B signaling through the loss of IKK proteins has also been linked to tumorigenesis, suggesting some tumor suppressive functions. These include genetic and chemically induced mouse tumor models and studies of squamous cell carcinomas of the skin, lungs, and head and neck [132, 133]. Interestingly, a recent report by Wang et al. suggests that NF*κ*B signaling may begin as a tumor suppressive pathway in mouse embryonic fibroblasts (MEFs), by promoting cell senescence and maintaining genome stability, as determined using* p65* ^−/−^ MEFs. NF*κ*B signaling can then switch to a tumor promoting pathway as cells undergo transformation, such as the introduction of mutant Ras^G12V^ into the MEFs, by allowing the transformed cells to avoid macrophage-induced cell death and evading other antitumor immunity activities* in vivo* [134]. Thus, the role of NF*κ*B signaling in tumorigenesis is complex and dynamic.

DEK has been identified as a downstream target of noncanonical NF*κ*B signaling. In normal human dermal fibroblasts, the loss of noncanonical pathway members NF*κ*B2 and RelB by siRNA resulted in decreased* DEK* mRNA and protein levels, which was associated with cellular senescence, in a p53-dependent mechanism [135]. The loss of p53 by shRNA restored DEK expression in NF*κ*B2 and RelB-deficient cells and prevented senescence induced by the DEK depletion [135]. This suggests that p53 and noncanonical NF*κ*B signaling converge on the* DEK* promoter to decide cell fate.

Previous reports have demonstrated that DEK can function as both transcription factor coactivator and corepressor [33, 125, 136–138]. Sammons et al. were the first to report on DEK-mediated regulation of canonical NF*κ*B signaling using MEFs, HEK293T, and HeLa cells. It was found that *Dek* ^−/−^ MEFs had elevated baseline and TNF*α*-induced levels of the NF*κ*B inhibitor, I*κ*B*α*, although the phosphorylation status of I*κ*B*α* was not investigated. *Dek* ^−/−^ MEFs also had increased luciferase expression from a NF*κ*B-reporter construct and enhanced TNF*α*-induced transcription of the NF*κ*B target gene and inflammatory chemokine* monocyte chemoattractant protein-1* (*MCP-1/CCL-2*). Furthermore, *Dek* ^−/−^ MEFs demonstrated increased TNF*α*-induced p65 (RelA) localization to the* MCP-1 *and* IκBα* promoters [125].

In transformed cells, including Caski and HeLa cervical carcinomas, the loss of DEK by shRNA caused increased phosphorylation of I*κ*B*α*. This was accompanied by the subsequent nuclear translocation and DNA-binding of p65 and increased luciferase reporter activity [124, 139]. Furthermore, in HeLa cells, DEK and p65 colocalized to multiple NF*κ*B target gene promoters, including* 1-cys-peroxiredoxin*,* c-IAP2*, and* IL-8* [125, 139]. Interestingly, TNF*α* treatment induced endogenous DEK-p65 colocalization at* c-IAP2* and* IL-8* promoters. This was accompanied by an increase in* c-IAP2* and* IL-8* mRNA levels [125]. However, when DEK was overexpressed in HeLa cells, there was a gradual dose-dependent inhibition of p65 transcriptional activity [125, 139]. In particular, overexpression of the C-terminal DEK DNA binding domain demonstrated inhibitory activity based on* 1-cys-peroxiredoxin *luciferase reporter activity. In contrast, overexpression of the N-terminal 200 amino acids of DEK, which includes the SAP/ΨSAP DNA binding domains, were capable of activating reporter expression [139]. It is worth mentioning that there is a dose-effect observed with DEK expression and cell viability. Optimal cellular proliferation is observed at DEK levels slightly (2–5 fold) over those observed in normal cells, similar to the endogenous levels of DEK in HeLa cells. Both the loss of DEK by shRNA and the gross overexpression of DEK are detrimental and cause caspase-dependent apoptosis [38, 40–42, 140, 141] (and data not shown). Since the mechanism of apoptosis induced by extreme changes in DEK expression is unclear, studies regarding the activity of specific transcription factors in response to DEK expression levels should be approached with caution. However, the data still supports a role for DEK in modulating RelA transcriptional activity in canonical NF*κ*B signaling.

**Figure 1:**
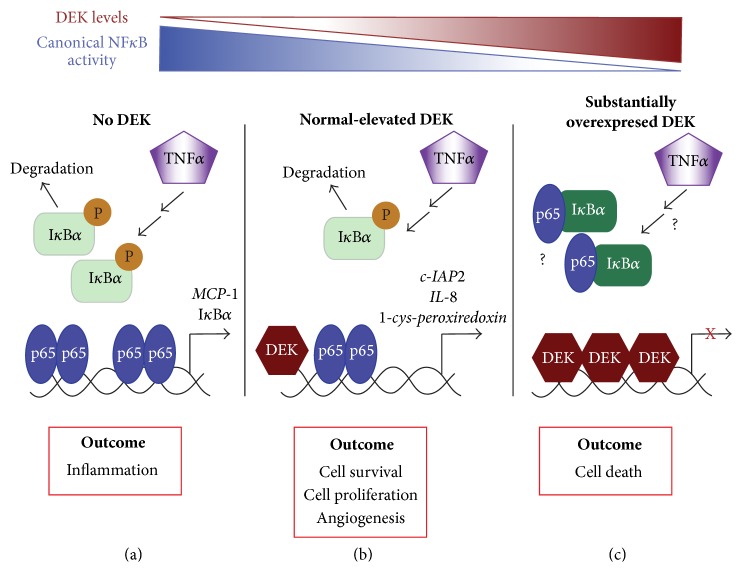
DEK-mediated regulation of NF*κ*B pathway activity in a dose-dependent manner. As DEK expression increases (red), canonical NF*κ*B transcriptional activity decreases (blue). (a) In the absence of DEK, there is increased phosphorylation of I*κ*Ba, which leads to its degradation and the translocation of p65/RelA to the nucleus where it is transcribes proinflammatory target genes like* MCP-1*. (b) In cells with endogenous levels of DEK, possibly in both normal and transformed cells, DEK colocalization with p65 on target gene promoters is induced by TNF*α* treatment. This results in the expression of potentially oncogenic, prosurvival target genes like* c-IAP2*. However, it is unknown how variations in DEK levels within this group, such as the difference between normal and transformed cells, may impact NF*κ*B activity and the subsequent expression of various target genes. (c) When DEK is substantially overexpressed beyond physiological levels, such as what may occur when overexpressing DEK in already high-expressing transformed cell lines, NF*κ*B activity is inhibited, which may trigger cell death.

Combined, the data suggest that DEK may provide a dose-dependent mechanism for controlling p65/RelA transcriptional activity in the canonical NF*κ*B pathway (Figure 1). In extreme excess, DEK inhibits NF*κ*B signaling, which may lead to decreased survival. However, tumorigenic (modestly upregulated) levels of DEK may promote NF*κ*B transcriptional activity through direct interactions with p65/RelA on gene promoters to induce expression of antiapoptotic genes like* c-IAP2 *and prometastasis genes like* IL-8*. In contrast, the loss of DEK can upregulate NF*κ*B activity through upstream regulation of I*κ*B*α*, which may correlate with an inflammatory or immune response as suggested by* MCP-1* expression. Taken together, DEK is an important regulator of NF*κ*B signaling to direct expression of both tumorigenic and proinflammatory target genes.

## 7. Summary

There is a growing understanding of the complex relationship between the immune system, inflammation, and tumorigenesis. Chronic inflammation is a well-known risk factor for tumor development, especially with epithelial tissues. This is likely due to either the highly oxidative environment during inflammation that can cause DNA damage and/or the creation of a highly vascularized growth factor-rich microenvironment resulting in a tumor-promoting stroma [142]. Various factors induce chronic inflammation including persistent bacterial and viral infections, exposure to environmental pollutants, and inflammatory autoimmune diseases. In addition to the creation of the tumor-promoting environment due to the immune response described above, the molecules produced by the immune system, like ROS and TNF*α*, can activate intracellular signaling pathways in the neighboring epithelial cells. One such example is the NF*κ*B pathway, which responds to inflammatory signals and promotes cell survival. When constitutively activated, such as what may occur with chronic inflammation, NF*κ*B signaling can promote tumorigenesis.

**Figure 2:**
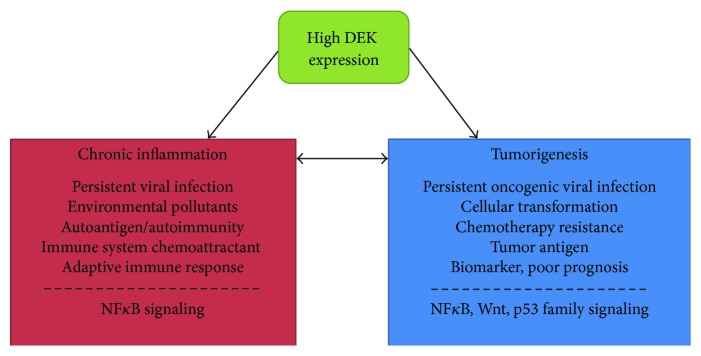
A summary of the roles of DEK in inflammation and tumorigenesis. DEK promotes inflammatory processes like persistent viral infection and autoimmune diseases, possibly through NF*κ*B signaling (left). High DEK levels also promote tumor growth, metastasis, chemotherapeutic response, and an overall poor prognosis, which correlates with the activity of many oncogenic molecular mechanisms including Wnt/*β*-catenin, mTOR, Rho, and NF*κ*B signaling as well as regulating expression of p53 family members (right). Chronic inflammation, supported by DEK expression, may promote tumorigenesis.

Here, we describe an oncogenic protein that is critically involved in infection, inflammation, and tumorigenesis. The chromatin modeling DEK protein has numerous roles that promote inflammation. These include (1) promoting the life cycles and latent infection with oncogenic viruses like HPV and EBV, (2) increased expression upon exposure to environmental pollutants, potentially to promote DNA repair or cell survival, (3) being a potent self-antigen in chronic inflammatory autoimmune diseases like arthritis and lupus, and (4) functioning as a proinflammatory chemoattractant to promote the migration of white blood cells when secreted by activated macrophages. These functions, whether they result in DEK overexpression or the internalization of excess secreted DEK by neighboring epithelial cells, can promote DEK-induced tumorigenesis (Figure 2). Elevated intracellular DEK levels are oncogenic, resulting in increased proliferation, migration, and resistance to genotoxic agents via the perturbation of several signal transduction pathways. It is unclear how DEK mediates these pathogenic and oncogenic cellular and molecular functions, although it is likely due, in part, to its ability to alter chromatin accessibility and transcription factor function, thus deregulating the expression of numerous target genes. The downstream pathways that are affected by DEK protein levels and DEK-induced transcriptional deregulation include p53, Wnt/*β*-catenin, mTor, Rho, and NF*κ*B signaling. Importantly, DEK can modulate the transcriptional activity of the NF*κ*B pathway in response to proinflammatory signals like TNF*α* in what may be a dose- or context-specific mechanism. Combined, DEK can promote both chronic inflammation and tumorigenesis in a multifaceted manner and has been implicated in numerous disease processes. This suggests that limiting DEK levels may be a desirable way to treat both chronic inflammation, due to viral infection or autoimmune disease, and cancer.
